# Submaximal loading as a feasible alternative to supramaximal loading in accentuated eccentric loading during the short-term basic strength block

**DOI:** 10.1371/journal.pone.0333613

**Published:** 2025-10-10

**Authors:** Caizheng Yue, Chunxiao Jia, Wenzheng Zha, Chenyu Zhang, Haokai Xu, Zhao Li

**Affiliations:** 1 School of Strength and Conditioning Training, Beijing Sport University, Beijing, China; 2 School of sport science, Beijing Sport University, Beijing, China; Università degli Studi di Milano: Universita degli Studi di Milano, ITALY

## Abstract

**Purpose:**

The purpose of this study was to compare the effects of supramaximal and submaximal accentuated eccentric loading (AEL) on lower-body function and hypertrophy during a short-term basic strength block.

**Methods:**

Twenty-two trained male students (20.64 ± 1.92 years, 177.07 ± 4.17 cm, 73.08 ± 4.44 kg) were assigned to two experimental groups based on baseline strength: the supramaximal loading group (SUPRA, 120%/70% one repetition maximum [1RM], *n* = 11) and the submaximal loading group (SUB, 95%/70% 1RM, *n* = 11), both groups applying eccentric overload during each repetition. Both groups trained twice weekly for 4 weeks (3 sets × 5 reps). The pre- and post-tests included rectus femoris cross-sectional area (RFCSA), countermovement jump height (CMJH), back squat 1RM, and 50% 1RM squat failure volume load (SFVL).

**Results:**

A statistically significant main effect of time was observed for all variables (*P* < 0.05), but no statistically significant group × time interaction effects were found for any variable (*P* > 0.05). Both the SUPRA and SUB showed no statistically significant improvements in RFCSA, with trivial changes observed (*P* > 0.05, Hedges’ g = 0.13 and 0.10, respectively). Both the SUPRA and SUB showed statistically significant improvements in CMJH, 1RM, and SFVL (*P* < 0.05), with similar changes in CMJH (Hedges’ g = 0.31 and 0.22, respectively) and 1RM (Hedges’ g = 0.46 and 0.38, respectively). In SFVL, the SUPRA showed slightly greater improvements than the SUB (Hedges’ g = 0.43 and 0.19, respectively).

**Conclusions:**

The results indicate that AEL training is effective in short-term training, with supramaximal and submaximal AEL producing similar improvements in outcomes. Therefore, submaximal AEL can serve as a feasible alternative to supramaximal AEL for physically active and healthy practitioners in a short-term basic strength block.

## Introduction

Traditional resistance training typically involves a combination of eccentric and concentric contractions to activate and stimulate different phases of muscle action. Owing to the force-velocity characteristics of muscle [1,2] and a tendency for lower motor units recruitment [3,4] observed during eccentric contractions, greater loads can be handled during the eccentric muscle actions compared to the concentric ones [1–4]. In practical applications, coaches and athletes usually perform traditional resistance exercises with same loads applied during both the eccentric and concentric phases. This approach may limit the full stimulation of the eccentric phase and the effective utilization of its unique characteristics [5,6]. Therefore, a strategy that allows for greater loading during the eccentric phase may help promote more effective muscular adaptations.

Accentuated Eccentric Loading (AEL) is an alternative method that optimally avoids the negative work isolation and stimulates the eccentric muscles well. It refers to a specific programming strategy in which the eccentric loading exceeds the concentric loading, and the movements used require a coupling of eccentric and concentric actions [7]. Due to the higher absolute eccentric loading involved, AEL serves as an effective approach to apply additional stress to the muscles and connective tissues while maintaining the concentric stimulus [6]. The choice of devices for implementing AEL appears to depend on practical application and the desired training outcomes [7]. For example, weight releasers (WRs) are commonly used to implement AEL, particularly in barbell squat [8–12] and bench press exercises [13,14]. WRs not only enable the smooth removal of the eccentric load during the transition from the eccentric to the concentric phase with minimal impact on the user’s movement technique, but also achieve load standardization in barbell exercises [6,7].

In training applications, appropriate and standardized load configuration is essential for optimizing training outcomes. The approach of setting eccentric loads based on concentric maximum strength one repetition maximum (1RM) has been widely accepted and applied in training practice [7,15]. Throughout the literature, supramaximal loading (i.e., loads above 100% of the concentric 1RM) is possible to train with loads of 120% to 160% 1RM during the eccentric phase [16]. However, in AEL exercises, the recommended supramaximal loading is typically between 110% and 120% of concentric 1RM [15]. Submaximal loading (i.e., loads lower than 100% of the concentric 1RM) in AEL implementation strategies may include more commonly used movement patterns, especially in power training and acute intervention protocols [14,17,18].

In fact, supramaximal loading has been shown to enhance muscle strength and is one of the most commonly employed strategies in AEL applications [7]. It is typically regarded as an advanced training tactic [6], requiring a high level of proficiency in AEL as well as sufficient strength capacity from the trainee [19]. However, due to the additional eccentric load or unfamiliarity with AEL exercises, supramaximal AEL training may lead to increased post-exercise soreness and greater muscle damage [20,21]. Moreover, the potentiating effect of supramaximal AEL training is dependent on relative strength, which may limit its effectiveness in individuals with lower strength levels [19,22]. In addition, a study showed that coaches or practitioners generally remain cautious about using AEL due to the lack of clear information on its scientific implementation [23], and even more caution is required when using supramaximal AEL. These concerns highlight the importance of exploring whether submaximal AEL can serve as a more accessible and effective alternative with lower technical and physiological demands, particularly during the early stages of AEL training. Since submaximal loading are below 1RM and within the body’ s controllable range, they can provide sufficient eccentric stimulus for most individuals, thereby laying a safer and more stable foundation for transitioning to supramaximal AEL training.

Several short-term studies (4–5 weeks) targeting basic strength or strength-endurance have shown AEL to be more effective than traditional resistance training in improving or maintaining performance [24–27]. Douglas et al. found that a 4-week slow-tempo submaximal AEL phase enhanced strength and speed more effectively than traditional training, whereas the subsequent 4-week fast-tempo supramaximal AEL phase may have exceeded recovery capacity, resulting in less favorable outcomes than traditional methods [24]. In a 4-week strength-endurance block, McDowell et al. reported that supramaximal AEL better maintained the rate of isometric force development compared to traditional resistance training [25]. Additionally, Munger et al. found in their 5-week study that supramaximal AEL training yielded superior improvements in eccentric strength and countermovement jump height compared to traditional training, while both methods produced similar results in concentric strength [26].

In a longer-term intervention, Maroto-Izquierdo et al. observed that both supramaximal and submaximal AEL elicited similar adaptations in mass and function over a 10-week period, though supramaximal loading resulted in greater gains in 1RM [28]. However, current research evidence remains limited regarding the specific chronic adaptations induced by AEL training with different eccentric load intensities during short-term training periods. Therefore, the aim of this study was to compare the short-term effects of supramaximal and submaximal AEL on lower body strength during a 4-week training period, with the goal of providing practical insights for coaches and practitioners. Based on previous studies on the chronic effects of AEL [24–26,28], we propose the following research question: Can submaximal AEL induce similar muscular adaptations as supramaximal AEL during short-term basic strength training?

## Materials and methods

### Subjects

The sample size was calculated using the G*Power 3.1. To achieve an actual power of 0.807, the input parameters for the analysis were as follows: an estimated effect size of *f* = 0.25 based on previous studies [24], α = 0.05, power (1 – β) = 0.8, number of groups = 2, number of measurements = 2, correlation = 0.5, nonsphericity correction = 1, resulting in a required sample size of *n* = 34. However, due to time constraints and subjects availability, a total of 22 male undergraduate students majoring in physical education were recruited for this study. Their descriptive characteristics are presented in Table 1. The inclusion criteria were as follows: a relative back squat strength of at least 1.5 times body mass, good general health, regular exercise in the past 3 months, including at least one squat session per week, no musculoskeletal or other relevant sports injuries in the past year. The recruitment period for this study started on 18-10-2024 and ended on 15-12-2024. Prior to participation, all subjects provided written informed consent after being fully informed of the purpose and potential risks of the study. They also agreed to refrain from participating in any other resistance training throughout the intervention period. This study was approved by the Beijing sport university ethics committee (Approval number: 2024357H), and all procedures were conducted in accordance with the Declaration of Helsinki.

**Table 1:** Mean ± standard deviation (SD) of subjects’ basic information.

Variable	SUPRA (*n* = 11)	SUB (*n* = 11)
**Age (y)**	20.82 ± 1.94	20.45 ± 1.97
**Body height (cm)**	177.23 ± 4.38	176.91 ± 4.15
**Body mass (kg)**	72.33 ± 4.08	73.83 ± 4.84
**Resistance training experience (y)**	3.36 ± 1.12	3.64 ± 1.29
**1-RM to body mass (kg·kg**^**-1**^ **body mass)**	1.83 ± 0.14	1.78 ± 0.16

### Design

**Fig 1:**
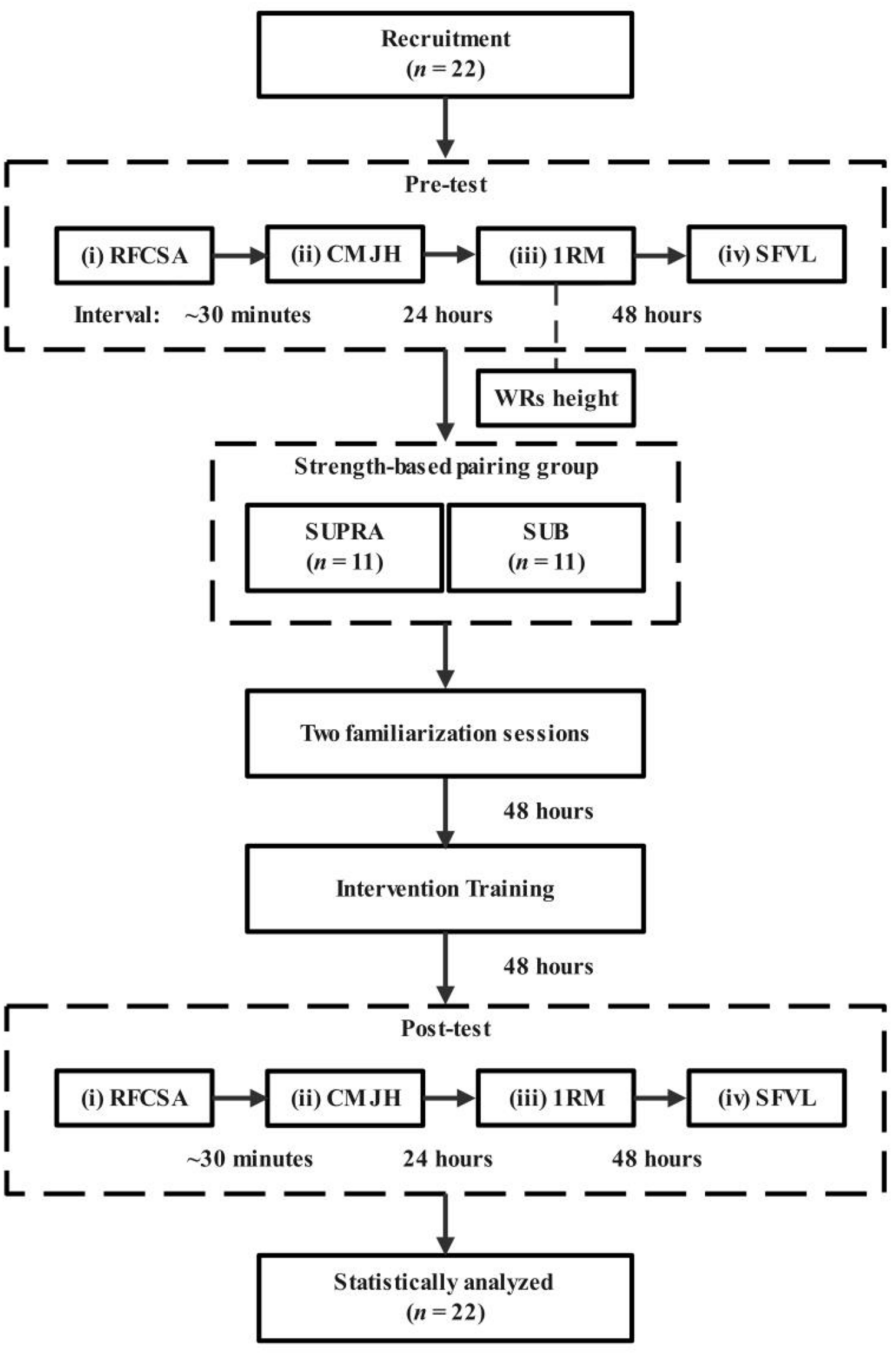
Experimental flowchart. The figure illustrates the entire experimental flow, including pretests, familiarization sessions, intervention training, and posttests. The height of WRs was determined after the completion of the 1RM test.

The aim of this study design was to compare the short-term effects of supramaximal and submaximal AEL on lower body function and hypertrophy during a 4-week training period, with the flow shown in Fig 1. Specifically, the subjects underwent the following assessments before and after the intervention: (i) rectus femoris cross-sectional area (RFCSA), (ii) countermovement jump height (CMJH), (iii) back squat 1RM, and (iv) squat failure volume load at 50% 1RM (SFVL). The testing sequence was as follows: RFCSA, CMJH, then 1RM testing, and finally SFVL testing. CMJH was performed after the RFCSA assessment, with at least 24 hours between the CMJH and squat 1RM tests, and at least 48 hours between the 1RM and SFVL tests. Notably, after completing the squat 1RM test, the height of the WRs was measured to ensure that they could disengage smoothly once the subject’s thigh was parallel to the ground. All subjects were instructed to avoid strenuous physical activity for 48 hours before the pre-tests and to maintain their usual diet and sleep routines. Additionally, subjects were paired based on their baseline 1RM strength levels and then randomly assigned to two experimental groups: the SUPRA group (eccentric: 120% 1RM, concentric: 70% 1RM, **n* *= 11) and the SUB group (eccentric: 95% 1RM, concentric: 70% 1RM, *n* = 11). All variables were homogeneous and normally distributed, with no significant baseline differences between groups (*P* > 0.05). Following the completion of the pretests, subjects performed two progressive familiarization sessions to get accustomed to the AEL protocol (Table 2). The intervention training commenced approximately 48 hours after the familiarization, and the posttests were conducted about 48 hours after the final training session. A standardized warm-up was conducted before all tests and training sessions, except for the RFCSA assessment.

**Table 2:** Familiarization sessions.

Familiarization	Group	WRs load	Barbell load	Set × repetition
**First**	All	15 kg	20 kg	2 × 6 ~ 8
		15% 1RM	40% 1RM	2 × 5
		25% 1RM	50% 1RM	1 × 3
		35% 1RM	60% 1RM	3 × 1
**Second**	All	0 kg (without WRs)	30% 1RM	1 × 5
		0 kg (without WRs)	50% 1RM	1 × 3
		40% 1RM	50% 1RM	1 × 2
	SUPRA	50% 1RM	70% 1RM	3 × 1
	SUB	25% 1RM	70% 1RM	3 × 1

### Training

In addition to the standardized warm-up, the intervention training also included a barbell-specific warm-up (Table 3). This warm-up was performed either with or without the use of WRs to adequately prepare the subjects for the subsequent training session. During the intervention training, at least two experienced experimenters manually attached the WRs to both sides of the barbell to ensure the smooth execution of the movement. Subjects performed a 2-second eccentric phase in response to a metronome cue, followed by the concentric phase as quickly as possible. Subjects were required to perform the squats continuously, with no more than a 3-second pause between repetitions. The eccentric loads for the SUPRA and SUB groups were set at 120% and 95% of concentric 1RM, respectively, while the concentric load for both groups was 70% of 1RM. Except for the difference in eccentric loading, all other resistance training variables were kept consistent between groups. Both groups followed a training protocol of 5 repetitions per set for 3 sets, with a 3-minute rest between sets, performed twice a week over a 4-week intervention period.

**Table 3:** Barbell warm-up procedure for the intervention training.

Group	WRs load (% 1RM)	Barbell load (% 1RM)	Set × repetition	Interset rest
**All**	0	30% 1RM	1 × 5	60 s
	0	50% 1RM	1 × 3	60 s
	40% 1RM	50% 1RM	1 × 2	180 s

### Hypertrophy

All measurements were conducted in the early morning, and subjects were instructed to avoid high-intensity training for 48 hours before testing. A qualified technician operated the ultrasound device (Vivid i, GE Healthcare, Wauwatosa, USA) for both pre- and post-tests to ensure consistency. To locate the measurement site for the rectus femoris, the distance from the anterior superior iliac spine to the superior border of the patella was measured using a vinyl measuring tape. One-third of this distance was marked from the distal end of the limb using a felt-tip pen to ensure consistent measurement across sessions. Subjects were instructed to lie in a supine position, remain still, keep their muscles relaxed, and breathe steadily during the measurements. The technician applied a water-soluble transmission gel to the measurement site, and an ultrasound probe (model: 12L) was placed perpendicular to the tissue interface without applying pressure to the skin. For all subjects, the measurement depth was set at 4 cm and the gain at 59 dB. Three images were captured, and the rectus femoris was outlined along the interface between the subcutaneous adipose tissue and muscle to determine its cross-sectional area (CSA). To ensure measurement accuracy, if the difference between the first two images was within 10%, their average was used. If the difference exceeded 10%, the third image was considered, and the average of the two closest values was used [29]. The test-retest intraclass correlation coefficient (ICC) was 0.994 (95% CI: 0.987–0.997).

### Explosive power

Explosive power was assessed using the countermovement jump test, conducted after the RFCSA measurement. The subjects’ maximum CMJH was recorded during three trials using dual portable force plates (Kunwei Force Platform, Kunwei Technology, Beijing, China). Prior to testing, all subjects received standardized instruction on proper jump technique. Subjects were instructed to keep their hands on their hips throughout the movement, with arm swinging not permitted and no restrictions on the depth. Each subject performed three trials, with one minute of rest between jumps, and the highest CMJH value was recorded for analysis. If a stable pre-jump force trace was not maintained, or if the subject’s hands moved away from their hips during the movement, the trial was repeated. The test-retest ICC was 0.970 (95% CI: 0.939–0.986).

### Muscle strength

Muscle strength tests were performed using free weights. Lower limb strength was assessed using the back squat 1RM. Before the test, subjects performed a 10–15-minute warm-up. They then performed the following squat sequence: 8 repetitions at approximately 50% 1RM with a 2-minute rest, 5 repetitions at approximately 70% 1RM with a 2-minute rest, 3 repetitions at approximately 80% 1RM with a 3-minute rest, and 1 repetition at approximately 90% 1RM with a 3- to 5-minute rest. Formal 1RM testing was then initiated, requiring subjects to descend to a depth where the top of the thigh was parallel to the floor. An attempt was considered unsuccessful if the participant failed to reach the required depth or was unable to complete the concentric phase with proper technique. Each trial was evaluated by at least two experimenters, and a repetition was classified as successful or failed only when both reached agreement. The experimenters gradually increased or adjusted the load, in accordance with previous completions, until 1RM was measured. All 1RM was assessed within 3–5 attempts.

### Muscle endurance

To minimize potential interference between muscle endurance and muscle strength testing, the muscle endurance test was scheduled to be conducted after the strength assessment [29]. Subjects performed parallel squats using a stationary smith machine to minimize movement variability and reduce muscle compensation. The load was set at 50% of each subject’s 1RM, determined from the baseline test for the pre-assessment and from the final 1RM for the post-assessment. A box was placed beneath the subject, adjusted to the height corresponding to their individual parallel squat depth. Subjects were required to initiate the concentric phase of the squat immediately after the posterior thigh made contact with the box. The squat cadence was set to normal, with approximately 2 seconds for the eccentric phase and 2 seconds for the concentric phase. The test was performed to volitional failure, which was defined as the inability to complete a repetition with proper technique, including any of the following criteria: (i) failure to reach the predetermined squat depth (i.e., loss of contact with the box), (ii) inability to maintain proper posture (e.g., excessive forward trunk lean), (iii) failure to complete the concentric phase, or (iv) inability to control the eccentric lowering tempo. The total number of successful repetitions was recorded by the experimenter, and muscle endurance was quantified as the total volume load, calculated as: 50% 1RM × number of repetitions completed to failure.

### Statistical analyses

All data were analyzed using IBM SPSS Statistics 29 software. Data normality was assessed using the Shapiro-Wilk test, and homogeneity of variance was assessed using Levene’s test. ICC estimates were calculated using a consistency, two-way mixed-effects model for reliability assessment. All main effects related to training were assessed by two-way repeated measures ANOVA (group × time). Paired t-tests were performed to assess within-group differences from baseline to post-intervention. The partial eta squared (*η*^2^) was used to estimate explained variance and effect size, interpreted as small (< 0.06), moderate (0.06–0.14), and large (> 0.14) effects [30]. Additionally, Hedges’ g was used to evaluate the effect size within each group. According to Hopkin’s guideline, the interpretation of Hedges’ g magnitudes is as follows: trivial, 0.00 to 0.19; small, 0.20 to 0.59; moderate, 0.60 to 1.19; large, 1.20 to 1.99; very large, ≥ 2.00 [31]. All values were expressed as mean ± SD. The two-tailed alpha was set at 0.05.

## Results

The results of the 4-week intervention training were shown in Table 4.

**Table 4:** Results of each measure at pre and post intervention training.

Measure	SUPRA			SUB			Time			Group × time		
	Pre	Post	Hedges’ g (CI)	Pre	Post	Hedges’ g (CI)	F	*P*	*η* ^2^	F	*P*	*η* ^2^
RFCSA (cm^2^)	7.26 ± 0.81	7.37 ± 0.81	0.13 (−0.68 to 0.93)	7.38 ± 0.75	7.45 ± 0.74	0.10 (−0.71 to 0.90)	5.343	0.032	0.211	0.137	0.715	0.007
CMJH (cm)	53.83 ± 3.99	55.11 ± 3.89	0.31 (−0.50 to 1.12)	52.11 ± 5.93	53.45 ± 5.76	0.22 (−0.59 to 1.03)	69.255	< 0.001	0.776	0.041	0.842	0.002
1RM (kg)	132.50 ± 13.60	138.64 ± 12.06	0.46 (−0.36 to 1.27)	130.91 ± 12.41	135.68 ± 11.46	0.38 (−0.43 to 1.19)	118.763	< 0.001	0.856	1.856	0.188	0.085
SFVL (kg)	1878.07 ± 270.53	1991.36 ± 242.10	0.43 (−0.40 to 1.23)	1791.02 ± 351.51	1860.91 ± 353.86	0.19 (−0.62 to 0.99)	38.312	< 0.001	0.657	2.151	0.158	0.097

Note: CI = 95% confidence interval.

### Hypertrophy

**Fig 2:**
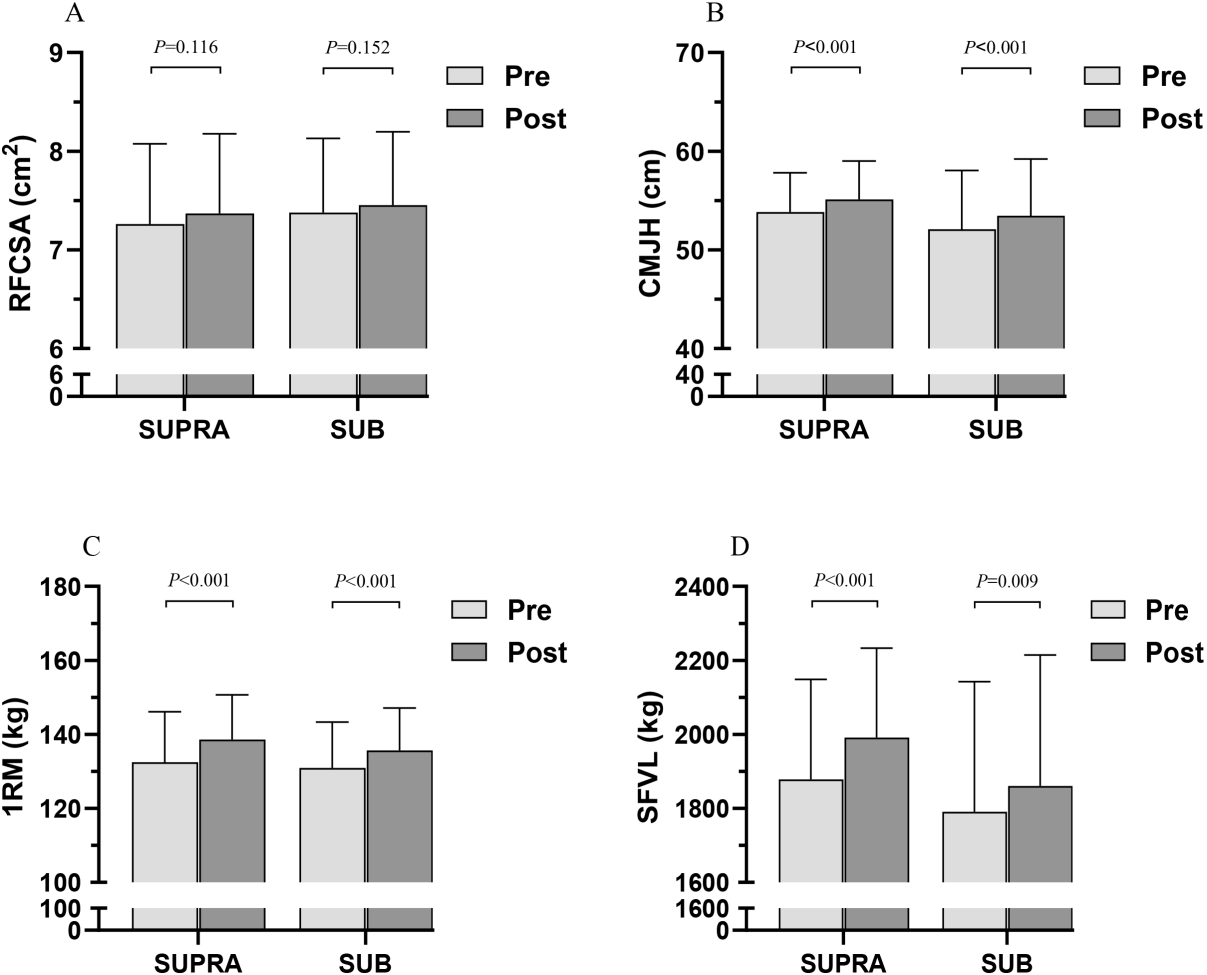
Comparison between the SUPRA and SUB groups at pre and post intervention training.

As shown in Table 4, a statistically significant main effect of time was observed for RFCSA (F = 5.343, *P* = 0.032, partial *η*^2^ = 0.211, large), while the group × time interaction was not statistically significant (F = 0.137, *P* = 0.715, partial *η*^2^ = 0.007, small). After the intervention training, ultrasound imaging of the rectus femoris muscle showed an increase in CSA, with increases of 0.11 ± 0.20 cm^2^ (Hedges’ g = 0.13, trivial) in the SUPRA group and 0.08 ± 0.16 cm^2^ (Hedges’ g = 0.10, trivial) in the SUB group. However, neither group showed statistically significant within-group differences from pre- to post-intervention in either group (**P* *> 0.05) (Fig 2A).

### Explosive power

After the 4-week intervention, a statistically significant main effect of time was observed for CMJH (F = 69.255, *P* < 0.001, partial *η*^2^ = 0.776, large). However, the group × time interaction for CMJH was not statistically significant (F = 0.041, *P* = 0.842, partial *η*^2^ = 0.002, small) (Table 4). After the 4-week intervention training, the SUPRA group showed an increase of 1.28 ± 0.50 cm (Hedges’ g = 0.31, small) in CMJH, while the SUB group increased by 1.35 ± 0.92 cm (Hedges’ g = 0.22, small). Statistically significant within-group differences were observed from pre- to post-intervention in both groups (*P* < 0.001) (Fig 2B).

### Muscle strength

A statistically significant main effect of time was observed for 1RM (F = 118.763, *P* < 0.001, partial *η*^2^ = 0.856, large), while the group × time interaction was not statistically significant (F = 1.856, *P* = 0.188, partial *η*^2^ = 0.085, moderate) (Table 4). After the intervention training, the SUPRA group showed an increase of 6.14 ± 2.34 kg (Hedges’ g = 0.46, small) in 1RM, while the SUB group increased by 4.77 ± 2.36 kg (Hedges’ g = 0.38, small). Statistically significant within-group differences were observed from pre- to post-intervention in both groups (*P* < 0.001) (Fig 2C).

### Muscle endurance

As shown in Table 4, a statistically significant main effect of time was observed for SFVL (F = 38.312, **P* *< 0.001, partial *η*^2^ = 0.657, large), while the group × time interaction was not statistically significant (F = 2.151, *P* = 0.158, partial *η*^2^ = 0.097, moderate). After the intervention training, the SUPRA group showed an increase of 113.30 ± 66.63 kg (Hedges’ g = 0.43, small) in SFVL, while the SUB group increased by 69.89 ± 72.07 kg (Hedges’ g = 0.19, trivial). Statistically significant within-group differences were observed from pre- to post-intervention in both the SUPRA (*P* < 0.001) and the SUB (*P* < 0.01) (Fig 2D).

## Discussion

This study aimed to compare the effects of supramaximal and submaximal accentuated eccentric loading AEL training on lower-limb function and muscle hypertrophy during a short-term basic strength block. After the 4-week intervention, all variables showed statistically significant main effects of time, but no significant group × time interaction effects were found. These findings suggest that AEL training is an effective intervention during a short-term basic strength block, with supramaximal and submaximal AEL producing similar improvements.

Previous studies have shown that AEL training can enhance muscular adaptations by increasing the CSA of fast muscle fibers, specifically type IIx and IIa fibers [32,33]. This may be attributed to growth factor- and myokine-induced muscle remodeling, activation of myoblast proliferation, or changes in the muscle proteome following AEL training, which may systematically enhance the anabolic environment and ultimately lead to an increase in CSA [34]. In a 10-week long-term intervention study by Maroto-Izquierdo et al., it was reported that although the supramaximal loading group (120%/30% 1RM) demonstrated superior improvements in 1RM strength compared to the submaximal loading group (90%/30% 1RM), both groups exhibited similar enhancements in anabolic responses and muscle hypertrophy [28]. Although previous studies have indicated that AEL can effectively enhance the anabolic conditions for muscle hypertrophy [33,35,36], an eccentric load difference of approximately 30% does not appear to result in a statistically significant difference between groups [28]. This study found statistically significant main effects of time for all variables, but no significant hypertrophic changes were observed from pre- to post-intervention in either AEL group, with trivial effect sizes (Hedges’ g = 0.10–0.13). This suggests that although the sample size may have limited the detection of significant within-group changes, the actual improvement in muscle hypertrophy was minimal. Given that the training phase in this study corresponded to a basic strength block, although the high eccentric load may have provided sufficient mechanical tension, the overall training volume was relatively low (5 repetitions per set, 3 sets in total), which may have been insufficient to induce the metabolic stress required for muscle hypertrophy. Additionally, the relatively wide CI observed in this study indicate considerable individual variability in response to short-term AEL training, which may be influenced by subjects’ strength levels and training experience. Furthermore, previous AEL studies targeting the elbow joint have indicated that significant muscle hypertrophy may not readily occur in trained individuals [37]. Therefore, we suggest that within a short-term intervention period, low-volume AEL training may be insufficient to induce significant muscle hypertrophy in trained individuals, and adaptations may vary greatly across individuals.

The results showed that both groups had significant improvements in 1RM after the intervention (Fig 2C). Given that muscle hypertrophy gains were trivial in both groups, it is likely that neural drive played a key role in strength development [38]. Specifically, AEL enhances muscle strength by activating the motor cortex [39], reducing spinal inhibition [40], and recruiting high-threshold motor units during muscle contractions [4]. In the study by Maroto-Izquierdo et al., the supramaximal loading group showed better improvements in maximum strength 1RM compared to the submaximal loading group [28]. Furthermore, studies have shown that with equal concentric loads, AEL exercises are more effective than traditional load training in improving maximum strength [7,41]. This suggests that higher eccentric loads may be more effective in enhancing maximum strength. However, in a 5-week progressive load intervention study by Yarrow et al., AEL training did not show better improvement than traditional loading training [42]. Additionally, in previously untrained individuals, AEL appears to yield similar strength gains as traditional loading training [43]. We believe that the effectiveness of AEL interventions appears to be influenced by the subjects’ strength levels, training protocol, intervention duration, and the ratio of eccentric to concentric loads [7,15,19,22]. Considering that the intervention in this study lasted only four weeks and involved approximately a 25% difference in loading, this may have been insufficient to produce a significant difference in 1RM improvement between the SUPRA and SUB groups (Table 4). Therefore, we suggest that for practitioners with limited AEL or strength training experience who aim to improve strength through AEL exercises in the short term, submaximal-intensity AEL may be a preferred and feasible option to consider.

When it comes to improvements in jump performance, optimization of the stretch-shortening cycle is believed to play a key role. In the present study, as shown in Fig 2B, both the SUPRA and SUB groups demonstrated significant within-group improvements in CMJH, with similar improvements (Table 4). Previous studies have indicated that AEL training can effectively enhance jump performance [24,33]. Douglas et al. reported that a 4-week AEL protocol involving 3-second controlled eccentric phases led to greater improvements in strength and maximal sprint speed compared to traditional resistance training [24]. These enhancements may be attributed to the distinct mechanical and neuromuscular mechanisms involved in AEL. In particular, during eccentric muscle elongation, the giant protein titin plays a critical role [44]. As additional eccentric loads are applied and subsequently released, increased titin stiffness may enable a more forceful and rapid recoil of muscle filaments, thereby enhancing subsequent concentric performance [45]. In AEL training, this mechanism also contributes to improved performance in stretch-shortening cycle movements through chronic adaptations [7]. Therefore, the improvements in jump performance observed in both groups may be attributed to enhanced stretch-shortening cycle function and strength optimization induced by eccentric overload. Nevertheless, the 25% difference in eccentric loading did not result in a significant difference in CMJH improvements between the two groups, suggesting that both loading strategies may be similarly effective in a short-term training.

Although high repetition, low load, and longer time under tension may be more prominent for muscle endurance development [29,46,47], our study showed statistically significant improvements in SFVL for both the SUPRA and SUB groups (Fig 2D). While the group × time interaction was not statistically significant, the SUPRA group appeared to have some advantage in improving SFVL (Hedges’ g = 0.43 vs. 0.19) (Table 4). Walker et al.’s study found that the number of repetitions to failure in knee extension improved only in the accentuated eccentric loading group, while no significant improvement was observed in the traditional training group [41]. Another study measuring the maximum repetitions in knee flexion indicated that AEL training effectively improves muscle endurance, and after the intervention, submaximal AEL training seemed to dilute the initial differences with supramaximal AEL training [28]. Both of these studies were based on long-term intervention training for unilateral knee maximum repetitions, while our study involved short-term intervention training based on pre- and post-test 1RM and total volume load of squat repetitions to failure at 50% 1RM. We believe that the improvement in 1RM seems to have contributed to the improvement in SFVL, as the total volume load improvement appears to be sensitive, even when the number of repetitions to failure remained unchanged.

This study has several limitations that should be considered when drawing conclusions from the findings. First, the absence of a traditional control group limits our ability to directly compare the short-term effects of AEL training with those of traditional resistance training. Second, the relatively small sample size may restrict the generalizability of the findings. Third, we only assessed strength and performance-related variables, lacking measurements of other physiological parameters or kinetic performance indicators. In addition, muscle hypertrophy was assessed solely based on the rectus femoris, which does not fully represent adaptations across the entire quadriceps muscle group [48]. Finally, all the study subjects were male undergraduate students with training experience. Therefore, the results of this study may not be applicable to all populations.

## Conclusion

In conclusion, our findings indicate that AEL training is effective in short-term training, with supramaximal and submaximal AEL producing similar improvements in outcomes. Therefore, submaximal AEL can serve as a feasible alternative to supramaximal AEL for physically active and healthy practitioners in a short-term basic strength block.

### Practical applications

The results of this study demonstrate the practical value of both supramaximal and submaximal AEL training within a short-term strength training block. Given that submaximal AEL shows comparable effectiveness to supramaximal AEL in the short term, it can serve as a safer and more accessible training option for coaches, strength and conditioning professionals, and athletes. Its relatively lower eccentric load helps reduce the risk of injury and excessive fatigue, making it particularly suitable for the early stages of AEL training or for individuals with limited experience in eccentric training. Moreover, submaximal AEL can facilitate technical skill acquisition and motor control development without compromising training effectiveness. Therefore, it may be used as a progressive strategy toward supramaximal AEL, helping practitioners to improve strength and performance in a safe and scalable manner.

## Supporting information

S1 File Raw data for all intervention variables included in the statistical analyses. (XLSX)
